# Relationships among athletes’ challenge and threat perceptions, mindfulness, and anxiety

**DOI:** 10.3389/fpsyg.2026.1811878

**Published:** 2026-03-17

**Authors:** Mehmet Bilgin Karademir, Melih Çalışır, Aydıner Birsin Yıldız, Ersan Tolukan, Şahinur Zararsız, Beyzanur Aydın, Doğukan Batur Alp Gülşen

**Affiliations:** 1 ALTSO Vocational School of Tourism, Alanya Alaaddin Keykubat University, Antalya, Türkiye; 2 Faculty of Sport Sciences, Bitlis Eren University, Bitlis, Türkiye; 3 Faculty of Sport Sciences, Ankara Yıldırım Beyazıt University, Ankara, Türkiye; 4 Institute of Health Sciences, Ankara Yıldırım Beyazıt University, Ankara, Türkiye; 5 Faculty of Sport Sciences, Aydın Adnan Menderes University, Aydın, Türkiye

**Keywords:** challenge perception, mindfulness, sport anxiety, athletes, threat perception

## Abstract

**Introduction:**

The aim of this study was to examine the associations between athletes’ challenge and threat perceptions and sport anxiety, and to evaluate the role of mindfulness in these relationships in terms of moderation and indirect effects (mediation).

**Methods:**

A total of 291 athletes from different sport branches participated in the study. Data were collected using the Challenge and Threat in Sport Scale, the Athlete Mindfulness Scale, and the Sport Anxiety Scale. Descriptive statistics and correlations were computed; regression-based models corresponding to PROCESS Model 1 were used for moderation analyses, and PROCESS Model 4 for indirect-effect analyses, with indirect effects evaluated using 5,000 bootstrap samples.

**Results:**

The findings indicated that mindfulness was negatively associated with sport anxiety, whereas threat perception was positively associated with sport anxiety. In the moderation models, the mindfulness × (challenge/threat) interaction terms were not significant, and the incremental contribution of the interaction to the models was negligible (ΔR² < .002). However, indirect-effect analyses showed significant indirect effects through mindfulness: for challenge → mindfulness → anxiety, the indirect effect was ab = −0.202 (95% CI [−0.300, −0.118]); and for threat → mindfulness → anxiety, the indirect effect was ab = 0.099 (95% CI [0.050, 0.160]).

**Discussion:**

Overall, the results suggest that mindfulness may function as a mechanism linking athletes’ cognitive appraisal processes to anxiety levels.

## 1 Introduction

The sport environment is inherently characterized by intense competition and a persistent pursuit of excellence. As such, it encompasses conditions that may be both facilitative and constraining for athletes. While competing under high performance expectations, athletes may sometimes perceive these conditions as opportunities for growth and challenge, whereas at other times they may interpret them as threatening situations focused on the possibility of failure (Kavussanu et al., 2021). In line with this perspective, the cognitive appraisal theory proposed by Lazarus and Folkman (1984) posits that individuals categorize encountered situations during the primary appraisal process as either a challenge or a threat, subsequently developing distinct coping strategies accordingly. Within this framework, the literature suggests that athletes’ responses to competition in sport settings primarily manifest in two forms: challenge and threat appraisals, as proposed in the Theory of Challenge and Threat States in Athletes (TCTSA) developed by Jones et al. (2009).

Challenge appraisal emerges when athletes believe they possess sufficient resources to meet situational demands and is typically associated with positive outcomes such as enhanced performance, motivation, and concentration. In contrast, threat appraisal arises when athletes evaluate situational demands as exceeding their perceived resources, and it is commonly linked to negative outcomes including performance decrements, anxiety, and heightened stress levels (Jones et al., 2009; Meijen et al., 2020; Robazza et al., 2024; Moore et al., 2012).

Researchers have indicated that when athletes experience threat appraisals, their attention tends to shift toward stimuli unrelated to the competitive task, stress responses intensify, and performance is adversely affected. Conversely, when competitive conditions are perceived as a challenge, athletes demonstrate more effective attentional focus on task-relevant cues and are better able to express their performance potential in a functional manner (Moore et al., 2012; Blascovich et al., 2003; Tomaka et al., 1997; Wright and Kirby, 2003). Comparative studies across different sport disciplines suggest that although athletes engage in similar cognitive appraisal processes, the orientation toward challenge or threat may vary depending on the type of sport. For instance, Cankurtaran (2021) reported statistically significant differences between sport branches in athletes’ challenge and threat appraisal scores, whereas Erman et al. (2023) found no significant differences based on whether athletes competed in individual or team sports. These findings suggest that challenge and threat appraisals are not solely determined by sport type but may also be closely related to individual athlete characteristics.

It has been reported that challenge and threat appraisals exert differential effects on attentional processes (Moore et al., 2013). In this context, one particularly important construct to consider is athletes’ mindfulness. Mindfulness is defined as the capacity to direct attention to the present moment in a nonjudgmental manner, while accepting one’s thoughts, emotions, and bodily sensations as they are (Kabat-Zinn, 2003). This approach is thought to support athletes in refocusing on task execution by acknowledging and accepting negative thoughts and emotions rather than struggling against them. Empirical evidence supports the notion that higher levels of mindfulness positively influence sport performance, reduce stress and anxiety, and enhance emotional regulation and psychological wellbeing (Brown and Ryan, 2003; Tasneem and Panwar, 2022; Gardner and Moore, 2017; Si et al., 2024; Dehghani et al., 2018).

Findings in the literature further indicate significant associations between mindfulness and challenge–threat appraisals. Athletes with higher mindfulness levels tend to report stronger challenge appraisals and lower threat appraisals, whereas those with lower mindfulness levels appear more prone to threat-oriented interpretations of competitive situations (Göncü and Balcı, 2023). Additionally, mindfulness has been shown to be negatively associated with threat appraisal (Solmaz and Yarayan, 2025) and anxiety levels (Parmentier et al., 2019). These findings suggest that mindfulness may play a regulatory role in how athletes evaluate competitive environments. Accordingly, mindfulness should be considered a key psychological resource influencing athletes’ challenge and threat appraisals.

Another fundamental psychological variable affecting performance in sport settings is anxiety. Anxiety is commonly defined as a negative psychological state arising from perceived stress when performing under pressure and is composed of both cognitive and somatic components. Although anxiety is considered a natural response to situations in which athletes’ abilities are evaluated, elevated anxiety levels may lead to attentional disruptions, reduced self-confidence, and performance impairments (Flett and Hewitt, 2005; García-Naveira, 2010; Küçükalpelli et al., 2025). Moreover, athletes with high anxiety levels are more likely to perceive competitive environments as threatening and to exhibit diminished challenge appraisals (Dias et al., 2011). Within this context, the literature indicates that athletes’ performance in competitive settings is closely related to their challenge and threat appraisals, mindfulness levels, and anxiety states. Evidence suggests that challenge and threat appraisals are associated with anxiety, that mindfulness is linked to both appraisals and anxiety, and that mindfulness and anxiety are themselves significantly related (Dias et al., 2011; Göncü and Balcı, 2023; Parmentier et al., 2019; Morrone et al., 2024; Moore et al., 2013).

When existing theoretical and empirical findings are considered collectively, it becomes evident that athletes’ performance is closely associated with their challenge and threat appraisals, mindfulness levels, and anxiety states (Kesler et al., 2026). Numerous studies have demonstrated that challenge and threat appraisals are related to anxiety, while mindfulness shows meaningful associations with both these appraisals and anxiety (Dias et al., 2011; Göncü and Balcı, 2023; Parmentier et al., 2019; Morrone et al., 2024). Accordingly, the purpose of the present study is to examine the relationship between challenge and threat appraisals and sport anxiety in athletes, and to evaluate the moderating and indirect roles of mindfulness within these relationships.

Beyond its potential buffering (moderating) role, mindfulness may also function as an explanatory mechanism linking cognitive appraisals to emotional outcomes. From a cognitive–affective perspective, appraisal processes influence emotional responses partly through attentional and regulatory mechanisms. Athletes who interpret competitive demands as challenges may exhibit greater present-moment awareness and acceptance, which in turn may reduce anxiety levels. Conversely, threat appraisals may undermine attentional regulation and increase anxiety. Therefore, it is theoretically plausible that mindfulness mediates the relationship between challenge–threat appraisals and sport anxiety.

The following hypotheses were tested in the present study:

*H1*: There is a negative relationship between athletes’ challenge appraisal and anxiety.

*H2*: There is a positive relationship between athletes’ threat appraisal and anxiety.

*H3*: There is a negative relationship between athletes’ mindfulness and anxiety.

*H4*: Mindfulness moderates the relationship between challenge appraisal and anxiety in athletes.

*H5*: Mindfulness moderates the relationship between threat appraisal and anxiety in athletes.

*H6*: Mindfulness mediates the relationship between challenge appraisal and sport anxiety.

*H7*: Mindfulness mediates the relationship between threat appraisal and sport anxiety.

## 2 Method

### 2.1 Sample and procedure

The present study employed a quantitative, cross-sectional, correlational design to examine the associations between athletes’ challenge and threat appraisals, mindfulness, and sport anxiety. Moderation hypotheses were tested using regression-based interaction models, and indirect-effect models were examined as additional analyses to explore potential process patterns among the variables. Given the cross-sectional nature of the data, findings are interpreted as relational rather than causal.

To determine the required sample size for achieving the research objectives, a power analysis based on multiple regression analysis was conducted using the G*Power software. The results indicated that, assuming a 95% statistical power (1–*β* = 0.95), a 5% significance level (*α* = 0.05), and a medium effect size (*f*^2^ = 0.10), a minimum of 113 participants was required to obtain statistically meaningful results. It should be noted that although G*Power is appropriate for estimating power in multiple regression models, Monte Carlo simulation is considered a more precise method for power estimation in mediation and moderation analyses. In the present study, indirect effects were tested using bootstrap procedures (5,000 resamples), which are recommended for mediation models and do not rely on normality assumptions. Furthermore, the non-significant moderation effects were associated with extremely small interaction effect sizes (*f*^2^ < 0.002), suggesting that the absence of moderation is more likely attributable to trivial interaction magnitudes rather than insufficient statistical power. Based on this analysis, and to account for potential data loss while increasing the robustness of the findings, it was aimed to recruit approximately three times the minimum required sample size.

The study sample included individuals who were actively competing as licensed athletes. Eligibility criteria required participants to provide voluntary informed consent and to complete all data collection instruments in full. Participants who failed to meet these criteria were excluded from the study. Individuals who did not regularly attend training sessions, did not hold an active sport license, or provided incomplete and/or inconsistent responses were not included in the analyses.

Participants were recruited using convenience sampling from athletes who voluntarily agreed to participate. A total of 291 athletes participated in the study. The mean age of the participants was 22.72 years (SD = 4.55, range = 18–42). Of the participants, 102 were female (35.1%) and 189 were male (64.9%). Regarding national-team status, 239 athletes were non-national (82.1%) and 52 athletes were national-team athletes (17.9%). The mean sport experience was 7.90 years (SD = 4.85, range = 1–32).

### 2.2 Measures

Data were collected using a Personal Information Form, the Challenge and Threat in Sport Scale, the Athlete Mindfulness Scale, and the Sport Anxiety Scale-2 (SAS-2).

The Personal Information Form, developed by the researchers, was used to collect demographic information such as age, gender, sport branch (individual/team), and years of sport participation.

The Challenge and Threat in Sport Scale was employed to assess athletes’ perceptions of competitive situations as either a challenge or a threat. The scale consists of 12 items and two subscales: *Challenge Appraisal* and *Threat Appraisal*. It is structured on a 6-point Likert scale. Turkish adaptation studies have reported satisfactory construct validity and acceptable internal consistency coefficients for both subscales. In the present study, separate total scores for the challenge and threat dimensions were used to represent the respective variables.

The Athlete Mindfulness Scale was used to assess athletes’ levels of mindfulness. The scale consists of 15 items across three subdimensions: *Awareness*, *Nonjudgment*, and *Refocusing*. It is rated on a 6-point Likert scale. The Turkish adaptation study confirmed adequate construct validity and acceptable internal consistency. In this study, the total score of the scale was used to represent the mindfulness variable.

The Sport Anxiety Scale-2 (SAS-2) was used to assess athletes’ multidimensional anxiety levels. The scale includes 15 items and three subdimensions: *Somatic Anxiety*, *Worry*, and *Concentration Disruption*. Items are rated on a 4-point Likert scale. Turkish validity and reliability studies have reported satisfactory psychometric properties. In the present study, the total score was used to represent sport anxiety.

### 2.3 Data analysis

Data analyses were conducted using IBM SPSS Statistics (Version 24). Prior to analysis, the dataset was screened to verify variable coding and the accuracy of composite score calculations (challenge appraisal, threat appraisal, mindfulness, and sport anxiety). The dataset was examined for missing values, and no missing observations were identified for the variables included in the analyses. Data cleaning and assumption testing were then performed.

Univariate outliers were evaluated using standardized *Z*-scores for continuous variables. Normality assumptions were assessed through skewness–kurtosis values and graphical methods (histograms and probability plots). Linearity and the suitability of error terms were examined based on regression outputs. Multicollinearity was evaluated using tolerance and variance inflation factor (VIF) values. Tolerance values exceeded 0.20 and VIF values were below 5 for all predictors, indicating no multicollinearity concerns.

Given that data were collected using self-report measures from a single source, the potential risk of common method bias was assessed using Harman’s single-factor test. The first factor accounted for 21.38% of the total variance, which was below the 50% threshold, suggesting that common method bias was not a serious concern.

The reliability of the measurement instruments was evaluated using Cronbach’s alpha internal consistency coefficients. Descriptive statistics (mean, standard deviation, minimum, and maximum values) were reported for all study variables. Pearson product–moment correlation coefficients were calculated to examine bivariate relationships among variables. The significance level was set at 0.05 for all analyses.

To test the main hypotheses and examine the moderating role of mindfulness, Hayes’ PROCESS macro was employed (Hayes, 2022). Moderation hypotheses were tested using two separate models. First, the moderating effect of mindfulness on the relationship between challenge appraisal and sport anxiety was examined (PROCESS Model 1; X = challenge appraisal, W = mindfulness, Y = sport anxiety). Second, the moderating effect of mindfulness on the relationship between threat appraisal and sport anxiety was tested (PROCESS Model 1; X = threat appraisal, W = mindfulness, Y = sport anxiety). Continuous variables were mean-centered to facilitate interpretation, and interaction terms (X × W) were generated using centered scores within PROCESS. Moderation effects were evaluated based on the significance of the interaction term coefficients.

As an additional analysis, indirect effect models were tested to examine potential mediational patterns among the variables, with mindfulness included as a mediator using PROCESS Model 4. Specifically, the pathways challenge appraisal → mindfulness → sport anxiety and threat appraisal → mindfulness → sport anxiety were examined. The significance of indirect effects was evaluated using 95% confidence intervals based on 5,000 bootstrap samples, with confidence intervals not including zero considered indicative of a significant indirect effect. In line with the theoretical focus of the study, analyses were conducted without including control variables, in order to assess the core relationships among the constructs.

The data used in this study were collected following ethical approval obtained from the Ankara Yıldırım Beyazıt University Rectorate Ethics Committee Coordination Board (Approval date: 19 September 2025; Approval no: 07/1429). After ethical approval was granted, prospective participants were informed about the purpose of the study, the voluntary nature of participation, and that the data would be used solely for scientific purposes. Participants who voluntarily agreed to take part in the study were asked to respond to all items completely and honestly. All stages of the research were conducted in accordance with the ethical principles of the Declaration of Helsinki issued by the World Medical Association.

## 3 Result

### 3.1 Reliability of the data

The internal consistency of the data used in the study was assessed using Cronbach’s alpha coefficients. For the Challenge dimension, the alpha coefficient was *α* = 0.828, while for the Threat dimension it was *α* = 0.875. Regarding the Athlete Mindfulness Scale, the alpha coefficients for the subdimensions were *α* = 0.741 for *Awareness*, *α* = 0.825 for *Nonjudgment*, and *α* = 0.781 for *Refocusing*, whereas the overall scale demonstrated an internal consistency coefficient of *α* = 0.695.

For the Sport Anxiety Scale-2, the alpha coefficients for the subdimensions were *α* = 0.736 for *Somatic Anxiety*, *α* = 0.796 for *Worry*, and *α* = 0.802 for *Concentration Disruption*. The overall internal consistency coefficient for the total scale score was *α* = 0.908.

In addition to confirmatory factor analysis, composite reliability (CR) and average variance extracted (AVE) values were calculated to further evaluate convergent validity. CR values for all subdimensions exceeded the recommended threshold of 0.70, indicating satisfactory internal consistency (Hair et al., 2019). Although AVE values for some subdimensions were slightly below the 0.50 criterion, the corresponding CR values were above 0.70. According to Fornell and Larcker (1981), convergent validity may still be considered adequate when CR exceeds 0.70 even if AVE is below 0.50.

In the present study, CR values ranged from 0.830 to 0.878 for the Challenge–Threat scale subdimensions (AVE = 0.497–0.513), from 0.745 to 0.831 for the Athlete Mindfulness subdimensions (AVE = 0.373–0.503), and from 0.775 to 0.904 for the Sport Anxiety subdimensions (AVE = 0.416–0.656). Discriminant validity was assessed using the Fornell–Larcker criterion. The square root of AVE (√AVE) for each construct was greater than the inter-construct correlations, supporting adequate discriminant validity among the study variables. In line with applied measurement recommendations, one standardized loading slightly below 0.50 (but above 0.40) was retained due to theoretical consistency and acceptable CR/AVE patterns.

### 3.2 Construct validity of the scales

To examine whether the original factor structures of the scales were retained within the present sample, confirmatory factor analyses (CFA) were conducted. For the Challenge and Threat in Sport Scale, the proposed two-factor structure *Challenge* (MT4, MT5, MT7, MT8, MT10) and *Threat* (MT1, MT2, MT3, MT6, MT9, MT11, MT12) demonstrated acceptable to moderate model fit (CFI = 0.905, TLI = 0.881, RMSEA = 0.097). Although the RMSEA value approached the upper threshold of acceptable fit, CFI values above 0.90 and the majority of standardized factor loadings exceeding 0.50 supported the adequacy of the two-factor structure. It has been noted that RMSEA may overestimate lack of fit in models with small degrees of freedom (Kenny et al., 2015). No post-hoc modifications were conducted in order to preserve the theoretical integrity of the original scale. The standardized factor loadings ranged from 0.60 to 0.83 for the Challenge dimension and from 0.43 to 0.81 for the Threat dimension, which is consistent with findings reported in previous adaptation studies. Although one loading was slightly below 0.50, it exceeded the 0.40 minimum often considered acceptable in applied measurement research, and the construct demonstrated adequate CR/AVE patterns; therefore, the item was retained to preserve theoretical content coverage.

The Athlete Mindfulness Scale exhibited acceptable model fit for its three-factor structure (CFI = 0.910, TLI = 0.891, RMSEA = 0.070). Standardized factor loadings ranged from 0.45 to 0.67 for *Awareness*, 0.57 to 0.88 for *Nonjudgment*, and 0.60 to 0.71 for *Refocusing*.

Similarly, the Sport Anxiety Scale-2 demonstrated good to acceptable fit for its three-factor structure (CFI = 0.938, TLI = 0.925, RMSEA = 0.072). Standardized factor loadings ranged from 0.43 to 0.76 for *Somatic Anxiety*, 0.71 to 0.91 for *Worry*, and 0.49 to 0.73 for *Concentration Disruption*.

The descriptive statistics for the study variables are presented in Table 1. Participants’ mean scores were 23.69 (SD = 4.96) for challenge appraisal, 21.41 (SD = 7.69) for threat appraisal, 62.07 (SD = 7.65) for mindfulness, and 28.91 (SD = 8.33) for sport anxiety. The skewness and kurtosis values were within the acceptable range for normal distribution as suggested by Tabachnick and Fidell (2013) (−1.5 to +1.5), indicating that the assumption of normality was satisfied for all variables.

**Table 1:** Descriptive statistics for the study variables.

Variable	Mean	SD	Min	Max	Cr *α*	Skewness	Kurtosis
Challenge appraisal	23.69	4.96	5	30	0.828	−0.777	0.779
Threat appraisal	21.41	7.69	7	42	0.875	−0.143	−0.489
Mindfulness	62.07	7.65	36	86	0.695	0.050	0.780
Sport anxiety	28.91	8.33	15	60	0.908	0.716	0.652

*p < 0.05, **p < 0.01, ***p < 0.001.

When the Pearson correlation analyses among the variables were examined, sport anxiety was found to be negatively associated with challenge appraisal (*r* = −0.182, *p* = 0.002) and mindfulness (*r* = −0.399, *p* < 0.001), and positively associated with threat appraisal (*r* = 0.390, *p* < 0.001). In addition, mindfulness was positively related to challenge appraisal (*r* = 0.317, *p* < 0.001) and negatively related to threat appraisal (*r* = −0.293, *p* < 0.001) (see Table 2).

**Table 2:** Correlations among the study variables.

Variable	Challenge appraisal	Threat appraisal	Mindfulness	Sport anxiety
Challenge appraisal	–	−0.046	0.317***	−0.182**
Threat appraisal		–	−0.293***	0.390***
Mindfulness			–	−0.399***
Sport anxiety				–

*p < 0.05, **p < 0.01, ***p < 0.001.

### 3.3 Moderation analyses

The moderating role of mindfulness was examined using regression-based moderation analysis. Continuous variables were mean-centered prior to analysis, and interaction terms were computed using the centered variables (see Table 3).

**Table 3:** Moderation model 1: X = challenge appraisal, W = mindfulness, Y = sport anxiety.

Predictor	*b*	SE	*t*	*p*
Challenge appraisal	−0.089	0.098	−0.90	0.367
Mindfulness	−0.416	0.062	−6.69	<0.001
Challenge appraisal × mindfulness	0.008	0.012	0.66	0.508

Model fit: *R*^2^ = 0.164, *F*(3, 287) = 18.70, *p* < 0.001; VIF = 1.05–1.17, tolerance = 0.854–0.949.

According to the results, Model 1 was statistically significant (*R*^2^ = 0.164). Within the model, mindfulness emerged as a significant negative predictor of sport anxiety (*b* = −0.416, *p* < 0.001). However, the main effect of challenge appraisal (*p* = 0.367) and the interaction between challenge appraisal and mindfulness (*b* = 0.008, *p* = 0.508) were not statistically significant. The incremental variance explained by the interaction term was minimal (Δ*R*^2^ = 0.00128), and the corresponding effect size was negligible (*f*^2^ = 0.00153) (see Table 4).

**Table 4:** Moderation model 2: X = threat appraisal, W = mindfulness, Y = sport anxiety.

Predictor	*b*	SE	*t*	*p*
Threat appraisal	0.323	0.058	5.55	<0.001
Mindfulness	−0.340	0.059	−5.80	<0.001
Threat appraisal × mindfulness	−0.003	0.007	−0.47	0.642

Model fit: *R*^2^ = 0.241, *F*(3, 287) = 30.40, *p* < 0.001; VIF = 1.00–1.10, tolerance = 0.910–0.996.

According to the results, Model 2 was also statistically significant (*R*^2^ = 0.241). Within this model, threat appraisal positively predicted sport anxiety (*b* = 0.323, *p* < 0.001), whereas mindfulness negatively predicted sport anxiety (*b* = −0.340, *p* < 0.001). However, the interaction between threat appraisal and mindfulness was not statistically significant (*b* = −0.003, *p* = 0.642). The incremental contribution of the interaction term to the model was minimal (Δ*R*^2^ = 0.00057), and the corresponding effect size was negligible (*f*^2^ = 0.00076).

Overall, the interaction terms contributed very little additional variance to the models (Δ*R*^2^ < 0.002), and therefore, the moderation hypotheses were not supported.

### 3.4 Indirect effect models

According to the results, the indirect effect models were tested using Hayes’ PROCESS macro (Model 4), and 95% bootstrap confidence intervals (CI) based on 5,000 resamples were reported. The findings indicated that the indirect effects via mindfulness were statistically significant for both challenge appraisal (ab = −0.202; BootSE = 0.0467; 95% CI [−0.303, −0.117]) and threat appraisal (ab = 0.099; BootSE = 0.0277; 95% CI [0.051, 0.158]) (see Table 5).

**Table 5:** Indirect effect models.

Model	a (X → mind.)	b (Mind. → Y|X)	c (Total effect)	c’ (Direct effect)	Indirect effect (ab)	Boot 95% CI (lower)	Boot 95% CI (upper)
Model 3 (X = challenge appraisal)	0.489 (<0.001)	−0.413 (<0.001)	−0.305 (0.002)	−0.103 (0.280)	−0.202	−0.303	−0.117
Model 4 (X = threat appraisal)	−0.291 (<0.001)	−0.339 (<0.001)	0.422 (<0.001)	0.324 (<0.001)	0.099	0.051	0.158

Values in parentheses indicate *p*-values. A bootstrap 95% confidence interval for the indirect effect that does not include zero indicates a statistically significant indirect effect.

Within this framework, Hypotheses H1, H2, and H3 were supported. Specifically, a negative relationship was observed between challenge appraisal and sport anxiety (*r* = −0.182, *p* = 0.002), a positive relationship between threat appraisal and sport anxiety (*r* = 0.390, *p* < 0.001), and a negative relationship between mindfulness and sport anxiety (*r* = −0.399, *p* < 0.001).

In contrast, Hypotheses H4 and H5 were not supported, as the interaction terms representing the moderating role of mindfulness were not statistically significant in either the challenge–anxiety (*b* = 0.008, *p* = 0.508) or threat–anxiety (*b* = −0.003, *p* = 0.642) relationships.

## 4 Discussion

The present study examined the relationships between challenge and threat appraisals and sport anxiety in athletes, as well as the role of mindfulness as both a moderator and a mediating mechanism within these relationships. The findings revealed a negative association between mindfulness and sport anxiety, indicating that higher levels of mindfulness are linked to lower anxiety levels in competitive sport contexts. This result is consistent with previous research demonstrating that mindfulness-based approaches are effective in reducing sport-related anxiety (Brown and Ryan, 2003; Mistretta et al., 2017).

The findings further indicated that sport anxiety was negatively associated with challenge appraisal and positively associated with threat appraisal. The negative relationship between challenge appraisal and anxiety aligns with theoretical perspectives suggesting that athletes experience lower anxiety when they perceive performance demands as manageable and within their coping resources (Kavussanu et al., 2014). Conversely, the positive association between threat appraisal and sport anxiety supports prior evidence indicating that evaluating competitive environments as overwhelming or uncontrollable increases anxiety levels (Lee et al., 2025; Martens et al., 1990).

The observation that mindfulness was positively related to challenge appraisal and negatively related to threat appraisal suggests that athletes’ ability to attend to the present moment without judgment may facilitate more functional cognitive appraisal processes. This finding is consistent with previous studies reporting that athletes with higher mindfulness levels are more likely to interpret competitive situations as opportunities for challenge rather than sources of threat (Göncü and Balcı, 2023; Robazza et al., 2024; Solmaz and Yarayan, 2025).

Regression analyses demonstrated that mindfulness negatively predicted sport anxiety, whereas threat appraisal positively predicted sport anxiety. These results reinforce the notion that mindfulness serves a protective function against anxiety and contributes to emotional regulation processes within sport settings (Dehghani et al., 2018; Yalçın et al., 2023).

However, the interaction terms representing the moderating role of mindfulness in the relationships between challenge–anxiety and threat–anxiety were not statistically significant. This finding suggests that mindfulness may function less as a contextual buffer (moderator) and more as an explanatory psychological mechanism linking cognitive appraisals to anxiety. Supporting this interpretation, the indirect effect analyses demonstrated that mindfulness constituted a significant mediating pathway in the relationships between both challenge and threat appraisals and sport anxiety. This pattern may be understood in light of the dispositional nature of the constructs measured in the present study. The mindfulness variable was assessed as a trait-based characteristic reflecting relatively stable individual differences rather than situational fluctuations. From a cognitive–affective perspective, appraisal processes influence emotional outcomes partly through attentional and regulatory mechanisms. In this context, trait mindfulness may function as an embedded regulatory component within the appraisal–emotion sequence rather than as a situational buffering mechanism that weakens the strength of relationships at different levels. Moderation effects are often more likely to emerge under state-dependent conditions, where psychological resources are dynamically activated in response to acute competitive stressors. Therefore, the absence of moderation in this study may reflect the stable, dispositional nature of mindfulness rather than the absence of a regulatory role. Future research employing state-level assessments during actual competitive events may provide clearer evidence regarding potential context-dependent moderation effects.

Although some studies have reported a moderating role of mindfulness (Aydın, 2021; Tubbs et al., 2018), such discrepancies may be attributable to differences in sample characteristics, outcome variables, and measurement instruments. In the present study, the predominantly trait-like nature of the variables may explain why mindfulness emerged as a process-oriented mediator rather than an interactional moderator.

Overall, the findings indicate that mindfulness, challenge appraisal, and threat appraisal play critical roles in shaping athletes’ sport anxiety. While mindfulness and challenge appraisal appear to reduce anxiety, threat appraisal exerts an anxiety-enhancing effect. Importantly, mindfulness seems to operate not as a moderating shield, but as a mechanism through which cognitive appraisals influence anxiety levels, highlighting the central role of cognitive evaluation processes in competitive sport environments.

## 5 Conclusion

The present study examined the relationships among challenge and threat appraisals, mindfulness, and sport anxiety in athletes. The findings revealed that sport anxiety was negatively associated with challenge appraisal and mindfulness, and positively associated with threat appraisal. While mindfulness did not moderate the relationships between appraisals and anxiety, it demonstrated significant indirect effects in both models.

Specifically, mindfulness statistically accounted for part of the association between challenge appraisal and anxiety and partially explained the relationship between threat appraisal and anxiety. These findings suggest that mindfulness may represent an explanatory pathway through which cognitive appraisal processes are linked to anxiety levels in competitive sport contexts.

Overall, the results contribute to a more integrated understanding of how appraisal processes and attentional awareness are related to athletes’ emotional experiences.

### 5.1 Practical implications

From an applied perspective, the findings highlight the importance of addressing both cognitive appraisal processes and attentional regulation mechanisms in competitive sport settings. Rather than focusing solely on general mindfulness training, coaches and sport psychologists may benefit from implementing targeted cognitive reappraisal strategies designed to reshape athletes’ interpretations of competitive demands.

For example, structured reflection sessions following high-pressure simulations may help athletes reinterpret physiological arousal as facilitative rather than debilitating. Emphasizing controllable performance factors and reinforcing athletes’ perceived coping resources may strengthen challenge-oriented evaluations while reducing threat appraisals. Brief appraisal-restructuring exercises integrated into pre-competition routines may further enhance adaptive emotional regulation.

In this sense, mindfulness-based approaches should not be conceptualized merely as broad stress-reduction techniques, but as part of a more comprehensive intervention framework that simultaneously targets attentional awareness and cognitive evaluation processes.

### 5.2 Future research and limitations

Future research should employ longitudinal and experimental designs to more thoroughly examine the causal effects of mindfulness on challenge and threat appraisals and their subsequent impact on anxiety. Studies incorporating diverse sport branches, performance levels, and age groups may further clarify the context-specific functions of mindfulness. Additionally, the use of state-based mindfulness measures may provide deeper insight into why moderation effects were not observed in the present study and help capture situational fluctuations in competitive contexts.

Several limitations should be acknowledged. First, due to the cross-sectional design, the indirect effects identified should be interpreted as relational patterns rather than evidence of causal mediation. Second, the analyses were conducted using regression-based observed scores, and potential measurement error may have influenced coefficient estimates and indirect effect magnitudes. Moreover, the exclusive use of self-report measures and the primarily trait-based nature of the constructs may have limited the detection of contextual (state-level) effects and moderation relationships. Therefore, the findings should be replicated and extended using alternative samples, longitudinal approaches, and experimental methodologies.

## Data Availability

The original contributions presented in the study are included in the article/supplementary material, further inquiries can be directed to the corresponding author.
